# Surgery for Aspergillus Empyema with Refractory Pleural Fistula Following COVID-19 Pneumonia after Temporizing Measure Using Endobronchial Watanabe Spigot: A Case Report

**DOI:** 10.70352/scrj.cr.24-0171

**Published:** 2025-07-10

**Authors:** Shota Umeda, Takahiro Nakajima, Osamu Araki, Takashi Inoue, Sumiko Maeda, Masayuki Chida

**Affiliations:** Department of General Thoracic Surgery, Dokkyo Medical University, Mibu, Tochigi, Japan

**Keywords:** COVID-19, refractory pulmonary fistula, invasive pulmonary aspergillosis, endobronchial Watanabe spigot

## Abstract

**INTRODUCTION:**

Approximately 20% of patients who contract coronavirus disease (COVID-19) pneumonia require oxygen therapy; of these patients, approximately 5% progress to acute respiratory distress syndrome, necessitating mechanical ventilation. The incidence of secondary infections among patients with COVID-19 is relatively low (16% for bacterial infections and 6.3% for fungal infections), but is predominantly observed in those with severe respiratory failure. Microvascular damage in COVID-19 can also lead to thrombus formation, causing infarctions, and in some cases, necrotizing pneumonia with cavity formation. Pulmonary resection may be necessary in patients who develop pneumothorax or empyema. Management options in complicated COVID-19 continue to evolve and should be individualized. Here, we present a case of Aspergillus empyema with refractory pleural fistula following COVID-19 pneumonia.

**CASE PRESENTATION:**

The patient was hospitalized in the intensive care unit for respiratory failure caused by COVID-19 pneumonia and developed a right pneumothorax 1 month after admission, with a halo sign in the middle lobe on computed tomography. Persistent massive air leakage and hypoxia developed, even with mechanical ventilation. Initially, to reduce the massive air leakage, endobronchial silicone spigot (endobronchial Watanabe spigot: EWS) were placed in the right B2 and middle lobe bronchi to stabilize the severe respiratory failure and septic shock. After EWS placement, the air leak decreased, with gradual improvement in the patient’s multi-organ failure status. Subsequently, the patient underwent a right middle lobectomy and upper lobe wedge resection. Histopathology confirmed an active Aspergillus infection in the resected lung, and voriconazole was administered postoperatively. Air leakage persisted postoperatively, necessitating repeat surgery and, finally, thoracoplasty and negative pressure wound therapy. The patient was eventually discharged with home oxygen therapy.

**CONCLUSIONS:**

This case illustrates the successful treatment of invasive pulmonary aspergillosis with refractory pulmonary fistula and empyema following COVID-19 pneumonia using a combination of endoscopic and surgical interventions. In cases of severe COVID-19 pneumonia, clinicians must remain vigilant for secondary infections, including aspergillosis. EWS placement can be effective in reducing significant air leakage and stabilizing patients’ condition.

## Abbreviations

ARDS
: acute respiratory distress syndrome
EWS
: endobronchial Watanabe spigot

## INTRODUCTION

During the clinical course of coronavirus disease (COVID-19) pneumonia caused by the severe acute respiratory syndrome coronavirus 2 (SARS-CoV-2) Delta variant, approximately 20% of patients require oxygen therapy, and approximately 5% progress to ARDS, eventually requiring mechanical ventilation. Secondary infections in patients with COVID-19 are relatively rare (16% for bacterial and 6.3% for fungal infections), primarily occurring in patients with severe respiratory failure. COVID-19 has also been reported to cause thrombosis due to microvascular damage, resulting in infarctions and, occasionally, necrotizing pneumonia with cavity formation. If complications such as pneumothorax or empyema develop, pulmonary resection may be required.

Although COVID-19 caused by the Omicron variant has been reported to be less severe, the virus continues to mutate, making it challenging to predict future virulence. Accordingly, management strategies for complicated COVID-19 are dynamic, continuing to evolve, and need to be tailored to each patient’s specific condition. Other virulent viruses, such as the H5N1 influenza virus, also pose significant risks, and surgical treatment may be necessary for severe respiratory infections complicated by viral pneumonia. Here, we present a case of Aspergillus empyema with refractory pleural fistula following COVID-19 pneumonia.

## CASE PRESENTATION

**Fig. 1:**
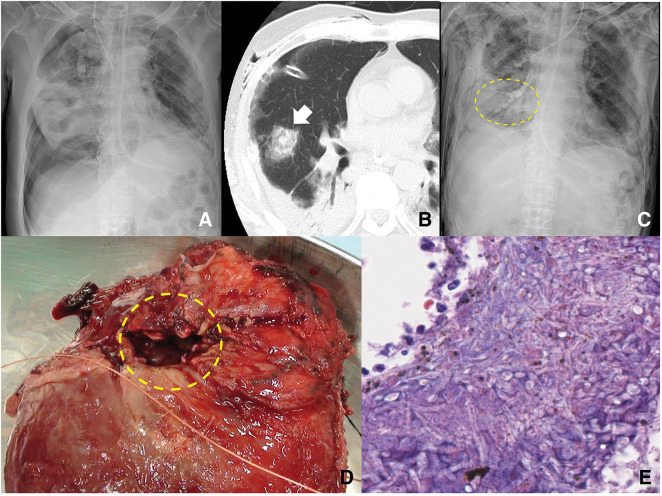
Patient’s clinical course. (**A**) Chest radiograph 1 month after COVID-19 pneumonia reveals a right pneumothorax. (**B**) Chest CT shows a halo sign in the middle lobe (arrow). (**C**) Chest radiograph shows the placement of EWS in the B2 and middle lobe bronchi (circle). After EWS placement, air leakage decreased and lung expansion improved. (**D**) Intraoperatively, a significant pleural defect in the middle lobe is observed (circle). The dropped EWS can be seen within the cavity. (**E**) Histological examination reveals Y-shaped, branching hyphae with sharp angles and no constrictions, indicating Aspergillus infection. EWS, endobronchial Watanabe spigot

A male patient in his 50s presented to the emergency department with fever, cough, and dyspnea. The patient was admitted with diabetic ketoacidosis and COVID-19 positivity confirmed via polymerase chain reaction. He was transferred to another hospital 3 days after stabilization but readmitted to our ICU due to respiratory failure caused by COVID-19 pneumonia. Despite treatment with remdesivir and dexamethasone, a right pneumothorax developed (**Fig. 1A**), necessitating insertion of a chest drain. CT revealed a halo sign in the right middle lobe (**Fig. 1B**). The patient showed a high level of β-D glucan (29.2 pg/mL) and serum positivity for Aspergillus antigen, suggesting fungal infection. We started antifungal treatment with micafungin and liposomal amphotericin B; however, the middle lobe lesion progressed to a cavitary lesion. Empyema subsequently developed due to a refractory pulmonary-pleural fistula in the right middle lobe. Positive-pressure ventilation was ineffective in resolving respiratory failure, and the patient was referred to our department for surgical intervention.

The patient presented in septic shock, with low blood pressure and peripheral circulatory failure. The massive air leakage necessitated hyperventilation, resulting in severe hypoxia and hypocapnia. To improve his condition, we decided to place EWS in the right B2b, B4a, B4b, B5a, and B5b bronchi (**Fig. 1C**) based on CT findings. After EWS placement, air leakage was significantly reduced, and the patient’s condition gradually improved. Subsequently, the patient underwent a right middle lobectomy (**Fig. 1D**) in the infection isolation area of the ICU. The bronchus was ligated with 1-0 silk and transected to avoid bronchial fistula. A large pleural defect was identified in the right middle lobe, and the thoracic cavity had severe adhesions. The abnormal tissue was extended to the upper lobe, which necessitated additional wedge resection of the S2 segment. Histopathologic examination revealed Aspergillus infection in the right middle lobe (**Fig. 1E**). Voriconazole was thus administered postoperatively.

**Fig. 2:**
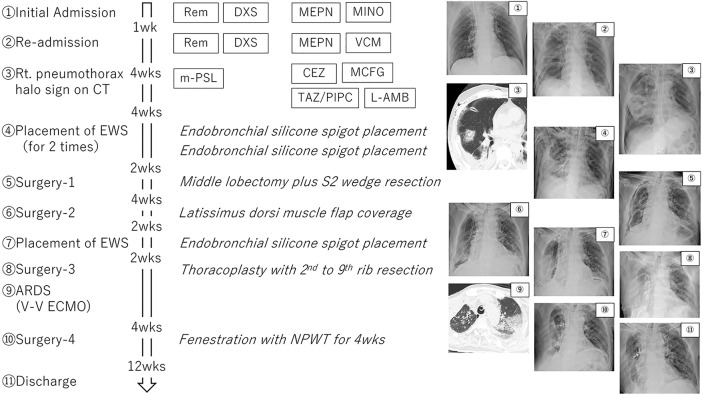
Clinical course of the case. ARDS, acute respiratory distress syndrome; CEZ, cefazolin; DXS, dexamethasone; EWS, endobronchial Watanabe spigot; L-AMB, liposomal amphotericin B; MCFG, micafungin; MEPN, meropenem; MINO, minocycline; m-PSL, methylprednisolone; NPWT, negative pressure wound therapy; Rem, remdesivir; TAZ/PIPC, tazobactam piperacillin hydrate; VCM, vancomycin; V-V ECMO, veno-venous extracorporeal membrane oxygenation; wk, week

One month postoperatively, the patient re-developed persistent air leakage and required repeat surgery with latissimus dorsi muscle flap coverage of the lung. Two weeks after the second surgery, air leakage recurred, and EWS was again placed to block the B2a and B3 bronchi. Finally, thoracoplasty was performed with 2nd to 9th rib resection. The patient developed acute respiratory distress postoperatively, requiring veno-venous extracorporeal membrane oxygenation support for 1 week. After recovery, localized empyema developed in the right thoracic cavity, and fenestration with negative pressure wound therapy was performed. On postoperative day 153 following this intensive treatment regimen, the patient was discharged with home oxygen therapy. The EWS were removed 1 year later, with no significant change in respiratory function. The patient has remained stable on home oxygen therapy at 2 L/min via a nasal cannula (**Fig. 2**).

## DISCUSSION

Mechanical ventilation is necessary in approximately 5% of patients with COVID-19 pneumonia who eventually develop ARDS. Secondary infections, particularly bacterial (16%) and fungal (6.3%) infections, are primarily observed in cases of severe respiratory failure.^1)^ Thrombus formation in COVID-19 occurs due to microvascular damage, leading to infarction and necrotizing cavitary pneumonia. In such cases, pneumothorax or empyema may develop, necessitating surgical intervention.^2–4)^

Secondary bacterial or fungal infections often occur approximately 10 days after hospitalization for severe COVID-19.^1)^ The combination of airway epithelial damage and lymphocyte reduction, particularly T-cell depletion, increases the risk of invasive fungal infections.^5)^ Invasive pulmonary aspergillosis develops in approximately 30% of patients with COVID-19 who develop ARDS, leading to a mortality rate as high as 64.7% (22 out of 34 patients).^6)^ *Aspergillus fumigatus* is the most common pathogen, for which voriconazole is the primary treatment; however, azole-resistant strains have been identified, necessitating careful drug selection. In this case, micafungin and liposomal amphotericin B were used preoperatively, with a shift to voriconazole postoperatively.^6)^

As for the second surgery for empyema, we could not use omentum owing to the severe adhesion of the right thoracic cavity around the right lower lobe and diaphragm. The efficacy of EWS in managing persistent air leakages has previously been demonstrated.^7)^ By placing the EWS in the affected bronchus, the airflow to the target segment is blocked, reducing air leaks. This approach is particularly valuable for high-risk patients who cannot tolerate surgery.^8)^ Similar bronchial occlusion strategies, such as spiration valve use for secondary pneumothorax in patients with COVID-19, have been reported.^9)^ In this case, we used EWS as a temporizing measure before surgery for invasive aspergillosis following COVID-19 infection, which resulted in successful management of the disease, allowing the patient to be discharged with home oxygen therapy despite the high mortality risk.

## CONCLUSIONS

This case demonstrates the successful treatment of invasive pulmonary aspergillosis with refractory pulmonary fistula and empyema following COVID-19 pneumonia using both endoscopic and surgical interventions. In cases of severe COVID-19 pneumonia, clinicians must remain vigilant for secondary infections, including aspergillosis. EWS placement can effectively reduce air leakage and stabilize patients with respiratory failure. In cases of surgery after COVID-19 pneumonia, the risk of postoperative respiratory failure is high, making early intervention and collaboration with intensivists crucial for successful outcomes.

## ACKNOWLEDGMENTS

We express our sincere gratitude to the ICU staff at Dokkyo Medical University Hospital for their dedicated support in managing this case.

## DECLARATIONS

### Funding

This case report did not receive any funding.

### Authors’ contributions

All authors have read and approved the final version of the manuscript and agree to be accountable for the work presented in this paper.

Shota Umeda: data collection, writing–original draft

Takahiro Nakajima: conceptualization, methodology, writing–review & editing

Osamu Araki: patient care, data curation

Takashi Inoue: patient care, data curation

Sumiko Maeda: patient care, data curation

Masayuki Chida: supervision, project administration.

### Availability of data and materials

The dataset supporting the conclusions of this article is included within the article.

### Ethics approval and consent to participate

The ethics committee of Dokkyo Medical University Hospital waived the requirement for ethical review due to the nature of this single case report.

### Consent for publication

The authors obtained written informed consent from the patient for the publication of this medical case report.

### Competing interests

Takahiro Nakajima received honoraria and lecture fees from Olympus Corporation. All other authors declare no conflict of interest related to this case report.
